# Supplementary material for: MetaRibo-Seq measures translation in microbiomes
Source: Nat Commun. 2020 Jun 29;11:3268. doi: 10.1038/s41467-020-17081-z (PMC7324362; doi:10.1038/s41467-020-17081-z)
Supplement: Supplementary file 10 — Supplementary Data 7 [file 41467_2020_17081_MOESM10_ESM.zip › File2/Confidence_High_Taxonomy/298107_out.krona.html]

Javascript must be enabled to view this page.

members
magnitude
magnitudeUnassigned
count
unassigned
taxon
rank

298107\_out

3

2
superkingdom
3

976
phylum
3

3
200643
class

order
171549
3

3
815
family

816
genus
3

1

SRS046712\_contig\_number\_124
species
818

2

SRS048060\_contig\_number\_2330SRS147377\_contig\_number\_9426
species
28116
